# The risk management of drowning in the Northern provinces of Iran

**Published:** 2019-07

**Authors:** Amran Pouraskariparast, Saeed Sadeghi

**Affiliations:** ^*a*^MA Student in Sport Management of Islamic Azad University, Eslamshahr, Tehran, Iran.; ^*b*^Associate Professor, Islamic Azad University, Eslamshahr, Tehran, Iran.

## Abstract

**Background:**

With the beginning of the hot weather and holiday season, the northern coast of the country is welcomed by many tourists to swim in the sea. However, the lack of proper management and safe facilities often lead to unpleasant incidents. Risk Management in Relief and Rescue is a responsibility to identify methods for dealing with potential threats that may have negative effects on swimmers' health.

**Methods:**

This applied descriptive study aimed at investigating the risk management of drowning in the coasts of Northern provinces. The statistical population consisted of all swimming places on the coasts of Guilan, Mazandaran and Golestan (n=560). Ninety-eight places were selected. A researcher-made questionnaire including age, sex and activity history, and another questionnaire including 6 subscales were used for assessing objective variables and risk management. The internal consistency of this questionnaire was obtained as α = 89 in same researches. Pearson correlation was used to test the data significance.

**Results:**

Life-saving and medical equipment, and personnel are not enough. The preservation and maintenance of rescue equipment, public culture on proper use of sea facilities, the status of training of the managers and rescuers and medical conditions were in poor condition, as well.

**Conclusions:**

Relief and rescue equipment in coastal provinces need to be increased quantitatively and qualitatively, and basic measures should be taken to improve the personal and public health in these areas. Paying attention and proper management in some primary issues can prevent the occurrence of drowning events. The appropriate measures included equipping survivors with rescue equipment and paying disbursement fees based on the salary table of the Life-saving Federation, informing the risks of swimming through the national media, and increasing the number of fixed and mobile relief and rescue posts on the beaches.

**Keywords:**

Risk Management, Educational Status, Environmental Health, Medical Conditions,Rescue and Relief

